# A Near Chromosome Assembly of the Dromedary Camel Genome

**DOI:** 10.3389/fgene.2019.00032

**Published:** 2019-02-05

**Authors:** Daniil Ruvinskiy, Denis M. Larkin, Marta Farré

**Affiliations:** ^1^Comparative Biomedical Sciences, Royal Veterinary College, University of London, London, United Kingdom; ^2^The Federal Research Center, Institute of Cytology and Genetics, Siberian Branch of the Russian Academy of Sciences, Novosibirsk, Russia; ^3^School of Biosciences, University of Kent, Canterbury, United Kingdom

**Keywords:** dromedary camel, genome, chromosome, assembly, camelids

## Abstract

The dromedary camel is an economically and socially important species of livestock in many parts of the world, being used for transport and the production of milk and meat. Much like cattle and horses, the camel may be found in industrial farming conditions as well as used in sporting. Camel racing is a multi-million dollar industry, with some specimens being valued at upward of 9.5 million USD. Despite its apparent value to humans, the dromedary camel is a neglected species in genomics. While cattle and other domesticated species have had much attention in terms of genome assembly, the camel has only been assembled to scaffold level, which does not give a clear indication of the order or chromosomal location of sequenced fragments. In this study, the Reference Assistant Chromosome Assembly (RACA) algorithm was implemented to use read-pair information of camel scaffolds, aligned with the cattle and human genomes in order to organize and orient these scaffolds in a near-chromosome level assembly. This method generated 72 large size fragments (N50 54.36 Mb). These predicted chromosome fragments (PCFs) were then compared with comparative maps of camel and cytogenetic map of alpaca chromosomes, allowing us to further upgrade the assembly. This dromedary camel assembly will be an invaluable tool to verify future camel assemblies generated with chromatin conformation or/and long read technologies. This study provides the first near-chromosome assembly of the dromedary camel, thus adding this economically important species to a growing pool of knowledge regarding the genome structure of domesticated livestock.

## Introduction

Dromedary camels (*Camelus dromedarius*) are members of the Camelidae family, the only family with extant species of the suborder Tylopoda, part of the Cetartiodactyla order. Camelids appeared ∼20 million years ago (Heintzman et al., 2015), and nowadays two main tribes of camelids exist, Old World camelids including the dromedary and Bactrian camel, and New World camelids with llamas, alpacas, vicunas, and guanacos. Camelids are characterized by karyotypes with a diploid number of 2*n* = 74 and almost identical chromosomes, with only slight variations in the amount and distribution patterns of heterochromatin (Balmus et al., 2007). Dromedary camels, as well as other camelid species, are adapted to harsh environments with dry, arid conditions and high temperatures (Gebreyohanes and Assen, 2017). Dromedary camels no longer exist in the wild; however, they are widely farmed in many countries with hot climates, such as Egypt, Syria, Libya, Somalia, Ethiopia, and Kazakhstan (Faye, 2015). Camels are not only used as means of transport, but also for dairy and meat production (Kebede et al., 2015). They are capable of producing milk for long periods of time and maintain its production under conditions where other animals would starve, thanks to having an unusually well-adapted udder for arid conditions (Alluwaimi et al., 2017). Although its economical and societal importance in developing countries, dromedary camel genomics has been understudied, and only recently, two dromedary camel genome assemblies were released (Wu et al., 2014; Fitak et al., 2016). However, both are assembled at scaffold level with an N50 of 4.1 and 1.40 Mb, respectively, making them unsuitable for in-depth use in evolutionary and applied genomics. To facilitate studies of genotype-to-phenotype associations for marker-assisted selection and breeding, high-quality chromosome-level assemblies are essential (Andersson and Georges, 2004). While such assemblies are established for popular livestock species, they are not available for those additional livestock species widely used in developing countries, including camels.

The African dromedary camel genome (Fitak et al., 2016) was assembled using next-generation sequencing (NGS) technologies. However, the use of short-read NGS data rarely produces assemblies at a similar level of integrity as those provided by traditional methodologies. NGS methods are incapable of generating long error-free contigs or scaffolds to cover chromosomes completely, requiring physical maps to upgrade NGS genomes to chromosome level (Lewin et al., 2009). Although new methodologies are being developed to overcome these limitations [e.g., long reads (Rhoads and Au, 2015), optical (Neely et al., 2011) or chromatin conformation maps (Lieberman-Aiden et al., 2009)], they often rely on hundreds of micrograms of high-molecular-weight DNA, which for some species are difficult to obtain, are usually expensive and suffer from misassembles. Bioinformatic approaches, e.g., the Reference-Assisted Chromosome Assembly (RACA) algorithm (Kim et al., 2013), were developed to approximate near chromosome-sized fragments for a *de novo* assembled NGS genome. RACA can assemble target genomes with no existing physical maps, utilizing their comparison to chromosome-level assemblies of reference and outgroup genomes, and read-pair data from target genome. RACA is suited for large, fragmented datasets such as the dromedary genome (Kim et al., 2013). Other reference-based algorithms e.g., RAGOUT (Kolmogorov et al., 2014) do not use the target assembly read-pair data to verify scaffold structures and orders, meaning that the target species-specific rearrangements could be missed from the reconstructed chromosome fragments, which could prove to be a problem in future candidate gene research, as a lower quality genome assembly will produce more false-negative and false-positive association signals, reducing the value of association studies (Goldfeder et al., 2016). Moreover, RACA has been successfully used for other genome assembly projects, including mammals [such as Tibetan antelope and red fox (Kim et al., 2013; Rando et al., 2018)] and birds [peregrine and saker falcons, ostrich, pigeon, and budgerigar (Damas et al., 2017; O’Connor et al., 2018)]. Finally, RACA assemblies could provide an independent source to prove and/or further improve assemblies produced with such methods as HiC, 10X or Dovetail Chicago.

In this report, therefore, we assembled the dromedary camel genome to near-chromosome level, using our previously established methodology (Damas et al., 2017). First, RACA was run to create predicted chromosome fragments (PCFs) and identify putatively chimeric scaffolds. These scaffolds could potentially contain structural errors and affect accuracy of PCFs or any other assemblies which would use them intact, therefore a subset of broken scaffolds was tested by polymerase chain reaction (PCR). Then, a second round of RACA was run to create a new, refined set of PCFs. And finally, taking advantage of the very stable camelid karyotypes (Balmus et al., 2007), we integrated previously published physical maps of dromedary camel (Balmus et al., 2007) and alpaca (Avila et al., 2014) to obtain a set of 72 chromosome fragments, with more than 80% of camel chromosomes assembled into three or less fragments. This new assembly will foster further genomic research into this special species and allow for improved genotype-to-phenotype studies.

## Materials and Methods

### Using the Reference Assisted Chromosome Assembly (RACA) to Assemble the Dromedary Camel Genome

Reference Assisted Chromosome Assembly was used to further assemble the dromedary camel genome into PCFs (Kim et al., 2013). As inputs, RACA took a target species’ (dromedary camel, Cdrom64K) scaffolds (Fitak et al., 2016), read-pair information, and the genome assemblies of a reference (cattle, bosTau6) and outgroup (human, hg19) species. The reference and outgroup species diverged 64.2 and 94.0 million years (MY) from camel, respectively.

#### Camel Read Sequence Data and Mapping

Sequence reads for dromedary camel (SRR2002493, SRR1950615, and SRR1693817) (Fitak et al., 2016) were downloaded from the National Center for Biotechnology Information (NCBI) using SRA toolkit v.2.8.2 (Leinonen et al., 2011). FastQC v.0.11.5 (Andrews, 2010) was used to evaluate the reads to decide on quality trimming. Bowtie2 v.2.3.0 (Langmead and Salzberg, 2012) was used to map camel reads to camel scaffolds, with insert minimum and maximum lengths of 250 and 750 bp for corresponding libraries (according to sequencing library information), trimming three base pairs from the 3′ end of each read.

#### Genome Alignments

To avoid spurious alignments, only original scaffolds longer than 10 Kb were used in this study. Lastz v.1.02.00 (Harris, 2007) was used for alignment of the camel scaffolds against the cattle assembly. Sequence alignments were concatenated into “chains,” which were then transformed into hierarchical “nets” alignments, according to alignment scores using Kent-library tools as described previously (Kent et al., 2003; Damas et al., 2017). The chain and net genome alignments between the human and cattle genomes were downloaded from the UCSC Genome Browser.

Reference Assistant Chromosome Assembly considers user-provided adjacencies of syntenic fragments (SFs) originating from different scaffolds as “reliable” and uses them to adjust read mapping thresholds. We defined reliable SF adjacencies *in silico*, using BLAT to map cattle genes to camel scaffolds. Cattle genes that mapped to two different SFs from two different camel scaffolds were then used as reliable SF adjacencies. These adjacencies were considered reliable, because if these SFs are not adjacent, the corresponding gene would need to be broken, which is unlikely due to high levels of gene conservation between mammalian genomes (Elsik et al., 2009).

#### RACA Run I

To improve the reliability of the final results, we ran RACA twice. Initially, the RACA algorithm was run to identify putatively chimeric scaffolds in the camel assembly, following our previous methodology (Farré et al., 2016). SFs were constructed at a 150 Kb resolution of SF detection, with default parameters except for: WINDOWSIZE = 10 and MIN_INTRACOV_PERC = 5.

#### PCR Testing of Putatively Chimeric Scaffolds

Primer pairs for testing putatively chimeric scaffolds were designed using Primer3 (v.2.3.6) (Untergasser et al., 2012) with optimum primer size of 20 bp (Supplementary Table S3). Only putatively chimeric scaffolds with a break interval size of <6 Kb were included in this analysis. Primers were chosen from camel sequences exhibiting high-quality alignments with the reference genome and the PCR product spanning the putatively chimeric join.

Camel DNA quality and concentration were tested using the Nanodrop 2000c (Thermo scientific). PCR was performed in a 10 μl volume with 5 μl Taq Polymerase Mix, 2 μl ddH_2_O, 1 μl of each primer at 2 μM in ddH_2_O and 1 μl of 30 ng/μl DNA solution. Thermal cycling was performed in the T100 thermal cycler (Bio-Rad) for 35 cycles: initial denaturation at 95°C for 3:00 min, 30 cycles of 95°C for 30 s (denaturation), 59–60°C at 1:00 min (annealing) and extension at 72°C at 1:00 min per PCR product 1,000 bp. Electrophoresis was done using the Sub Cell GT electrophoresis cell (Bio-Rad) with the power-pac basic power supply (Bio-Rad) with times ranging 20–40 min. PCR products were stained with SYBR-safe (Invitrogen) in a 1.5 and 1% agarose (Sigma) gel for PCR product lengths up to 2 and 4 Kb, respectively. Gels were visualized in a ChemiDOC MP system (Bio-Rad).

Polymerase chain reaction was done for two sets of primers per each putatively chimeric scaffold: the first set tested chimeric scaffold structure, and the second set tested the alternative (RACA-suggested) order of SFs from this scaffold, if a negative PCR result was observed for the first PCR following previous publication (Farré et al., 2016).

#### RACA Run II

Polymerase chain reaction confirmed non-chimeric scaffolds were included as an additional set of reliable SF adjacencies. The results of PCR testing also allowed to discern a physical coverage threshold of 212.5 read pairs (representing a coverage percentage of 51.16%), above which putatively chimeric scaffolds suggested by RACA are expected to be non-chimeric. As such, the second RACA was run with only one modified parameter: MIN_INTRACOV_PERC = 51.16. The results of the RACA run II were then transformed into a FASTA genome file, by joining the SFs in accordance with RACA’s instructions.

#### Evaluating PCFs and Assigning Them to Chromosomes

Predicted chromosome fragments obtained in RACA run II were manually compared with the fluorescence *in situ* hybridization (FISH) comparative map of the dromedary camel and human genomes (Balmus et al., 2007). The alignment output of camel PCFs to human chromosomes generated by RACA was used to verify and order PCFs along camel chromosomes. In addition to this, and making use of the highly stable camelid karyotypes, we compared the PCFs to a published cytogenetic map of alpaca (*Lama pacos*) (Avila et al., 2014). Coding sequences (CDSs) of the gene markers used in the alpaca map were downloaded from NCBI and mapped to dromedary camel PCFs using BLAT with default parameters. Only alignments spanning more than 80% of the CDS were considered reliable and analyzed further. PCFs with at least one marker were assigned to dromedary camel chromosomes following the alpaca gene map, while PCFs with at least two markers in the same order as in Avila et al. (2014) were placed and oriented into camel chromosomes (Avila et al., 2014).

Finally, the Benchmarking Universal Single-Copy Orthologs tool (BUSCO) (Simão et al., 2015) with the mammalian and laurasiatherian databases was used to verify completeness of core genes in the assembly. We then used REAPR (Hunt et al., 2013) to identify errors in our genome assembly without the need for a reference sequence with the short-insert size libraries.

## Results

Following our previous publication (Farré et al., 2016), our approach to assemble the dromedary camel genome to near-chromosome level involved three steps: (1) the construction of PCFs using the RACA algorithm; (2) PCR and computational verification of a subset of scaffolds that might contain species-specific chromosome structures or be chimeric; and (3) creation of a refined set of PCFs using the verified scaffolds and adjusted parameters to run RACA. We then used previously published physical maps of dromedary camel (Balmus et al., 2007) and alpaca (Avila et al., 2014) to verify the PCFs and assign them to dromedary camel chromosomes.

### Construction of PCFs From Scaffolds

A total of 4,922 camel scaffolds longer than 10 Kb, encompassing 1.99 Gb and representing 92.6% of the scaffold-based assembly, were aligned to cattle genome using lastZ and then concatenated to chains and nets as previously described (Kent et al., 2003). Overall, pair-wise alignments spanned 98.75 and 99.50% of cattle chromosomes for camel-cattle and human-cattle pairs, respectively. Five dromedary camel pair-end read libraries were mapped to camel scaffolds using Bowtie2 and the mapping coverage for each library was calculated using bedtools (Quinlan and Hall, 2010). Only three libraries (SRR2002493, SRR1950615, and SRR1693817) had an average coverage >17x of the camel genome and were used to run RACA.

An important input file to train RACA consists of SF adjacencies with a prior knowledge of being connected. To create this file, we made use of the high gene structure conservation in mammalian species (Elsik et al., 2009) and assumed that genes in one species are highly likely to maintain their structure in another closely related species. Therefore, we mapped cattle genes to camel scaffolds to identify genes aligned to two SFs containing two different camel scaffolds. A total of 23,819 cattle genes were used, of which 50 mapped to two different camel scaffolds, and were included as reliable adjacencies. Overall, the initial RACA run resulted in 73 PCFs with an N50 of 54.36 Mb covering 94.0% of scaffold-based assembly (Table 1).

**Table 1:** Statistics for RACA-based assembly of dromedary camel genome.

Statistics	Scaffold assembly	RACA run I	RACA run II
No. scaffolds	4,922	1,797	1,797
No. PCFs	NA	73	72
Homologous to complete reference chromosomes	NA	5	6
Total length (% of original assembly)	1,998,420,525 (100%)	1,886,430,396 (94.4%)	1,886,430,696 (94.4%)
N50 (Mb)	1.40	54.36	54.36
Max. length (bp)	9,719,801	122,837,232	122,837,232
Min. length (bp)	10,001	206,422	206,422
^∗^Max. no. scaffolds	NA	97	100
^∗^Min. no. scaffolds	NA	1	1
No. broken scaffolds	NA	46 (2.60%)	47 (2.62%)


*^∗^Min/max number of scaffolds are the minimum and maximum number of scaffolds represented in single PCFs.*

**FIGURE 1:**
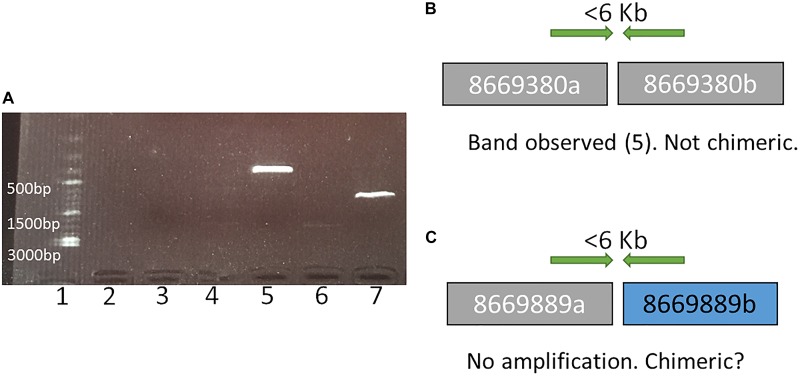
An example of representation and interpretation of the results of polymerase chain reaction (PCR) run I. **(A)** an electrophoresis gel, where in well 1 is a DNA ladder, 2 is the control (no DNA), 3 and 4 exhibit no PCR amplification for tested SF adjacencies in scaffolds 8669889 and 8669009, respectively, broken by RACA; 5 and 7 show PCR amplification for tested SF adjacencies in scaffolds 8669380 and 8669417, respectively, broken by RACA; 6 shows PCR product of unexpected size (2000 bp) for scaffold 8667696. **(B)** A schematic representation and interpretation of the PCR results from well 5 (scaffold 8669380, SFs 8669380a and 8669380b); **(C)** A schematic representation and interpretation of the PCR results from well 3 (scaffold 8669889, SFs 8669889a, and 8669889b).

**FIGURE 2:**
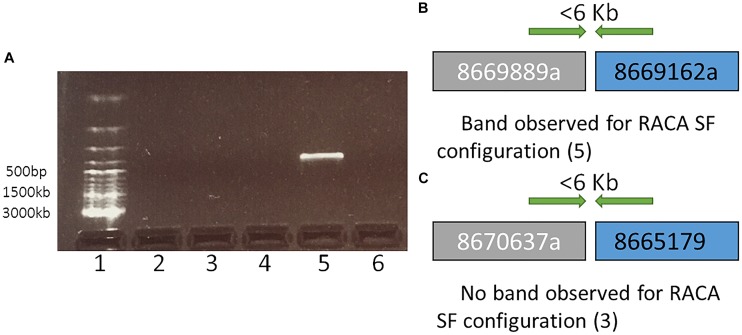
A representation and interpretation of the results of PCR run II. **(A)** An electrophoresis gel, where in well 1 is a DNA ladder; 2 is the control (no DNA); 3, 4, and 6 show no amplification for tested SF adjacencies 8670637a and 8665179, 8667696a and 8667368, 8669739a and 8669686, respectively. Order of these SF was suggested by RACA; 5 exhibits amplification for adjacent SFs 8669889a and 8669162b in the order suggested by RACA. **(B)** A schematic representation and interpretation of the results form well 5 (SFs 8669889a and 8669162b were confirmed to be adjacent suggesting that the original scaffolds 8669889 and 8669162 these two SFs originate from were chimeric); **(C)** A schematic representation and interpretation of the results from well 3. The order of SFs 8670637a and 8665179, adjacent in RACA’s output was not confirmed due to no PCR amplification, suggesting that no conclusion about the chimeric nature of scaffolds 8670637 and 8665179 could be made.

Reference Assistant Chromosome Assembly introduced 49 breaks in 46 (2.6%) camel scaffolds, and they were considered as putatively chimeric joints. These scaffolds contained structural differences from the cattle and human genomes, meaning that they could negatively affect PCF structures if proven to be chimeric. In order to assess these joints, primers were designed for 27 out of 49 putatively chimeric joints. A total of 14 of the 27 selected intervals resulted in PCR products of expected sizes, indicating that these joints were not chimeric (Figure 1, Table 2, and Supplementary Table S1). For joints with no amplification in PCR round I, we tested the alternative arrangements of SFs suggested by RACA (Figure 2 and Table 2). If the order of SFs suggested by RACA was confirmed by PCR, the corresponding scaffold(s) were classified as chimeric. This resulted in seven of 18 tested intervals being classified as chimeric (Table 2 and Supplementary Table S1). The reason there were more tested structures in the second run of PCR than there were negative results in the first run, is because there were two alternative SF arrangements that could be tested in the second PCR round (one per flanking SF) and for some scaffolds we tested both arrangements. Overall, seven scaffolds were confirmed as chimeric, while 14 were shown to be real. We could not make any conclusions regarding six scaffolds corresponding to 11 SF adjacencies (Table 2), because no PCR products were amplified in either of the rounds.

**Table 2:** Verification of putatively chimeric scaffolds by PCR.

Statistics	Camel
Pair-end read physical coverage within scaffolds	5.5 – 329.7
No. split SF adjacencies by RACA (default param.)	49
No. tested scaffold split regions	27
No. amplified split regions (confirmed SF joints)	14
No. non-amplified split regions	13
No. tested RACA-suggested adjacencies	18
No. amplified adjacencies (chimeric SF joints)	7
No. non-amplified adjacencies	11
Final no. ambiguous SF joints from tested split regions	11
Selected pair-end read spanning threshold	212.5
No. tested split regions found below selected threshold	22
No. chimeric SF joints	7
No. confirmed SF joints	4
No. ambiguous SF joints	11
No. tested split regions found above selected threshold	10
No. chimeric SF joints	0
No. confirmed SF joints	10
No. ambiguous SF joints	0


To estimate which of the remaining split scaffolds (>6 Kb or with ambiguous PCR results) were likely to be chimeric, we empirically identified a genome-wide minimum physical coverage (Meyerson et al., 2010) level in the SFs joining regions for which (and higher) the PCR results were consistent with RACA predictions. A physical coverage threshold of 212.5x was established, which would allow us to identify additional putatively chimeric scaffolds without any additional scaffold verification (Table 2 and Supplementary Table S1).

### Construction of a Refined Set of PCFs

**FIGURE 3:**
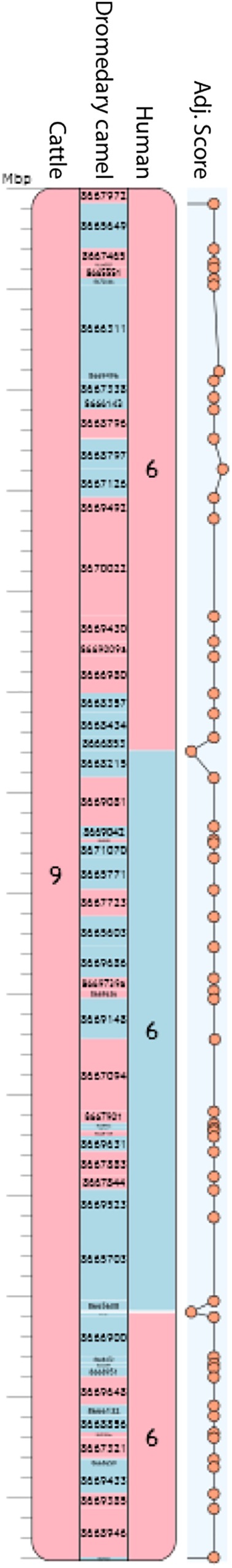
Camel chromosome 8 corresponding to PCF 9. Blue blocks indicate positive (+) orientation of tracks compared with the camel chromosome while red blocks, negative (-) orientation. Numbers inside each block represent cattle and human chromosomes or dromedary scaffold IDs. Adjacency scores are shown on the right-hand side of the PCF. The rest of the chromosomes can be found in Supplementary Figure S1.

Polymerase chain reaction-verified scaffolds, confirmed as non-chimeric but with a physical coverage below the new set threshold were used as additional reliable adjacencies for RACA run II. This run resulted in a final set of 72 PCFs with an N50 of 54.36 Mb (Table 1). The total length of the RACA assembly was ∼1.89 Gb. The longest PCF spanned 122.84 Mb and included 74 scaffolds, while the shortest was 206 Kb in size, containing only one scaffold. Six PCFs were homologous to complete cattle chromosomes (BTA9, BTA12, BTA19, BTA24, BTA25, and BTA27; Figure 3), from which only one (BTA19) showed an intrachromosomal rearrangement between cattle and dromedary genomes. A total of 46 scaffolds, representing 2.6% of scaffolds used by RACA, were still split despite some being present in reliable adjacencies.

### Assessment of PCFs With Dromedary and Alpaca Cytogenetic Maps and Generation of a Final Chromosome Level Assembly

In order to verify the RACA assembly, we compared our PCFs to previously published physical maps for dromedary camel and alpaca. First, PCFs mapping to two or more human chromosomes were compared to the dromedary camel-human cytogenetic map (Balmus et al., 2007). A total of 61 PCFs, representing 87.8% of the total assembled genome, agreed with FISH, while six PCFs (12.2% of assembled genome) presented disagreements. Four of the PCFs that disagree with FISH data (PCFs 2a, 8b, 7a_10_20a and 10e_21a) contained a small fragment (<3 Mb of size) mapping to a human chromosome not revealed by FISH. However, these PCFs might be correct, since the sizes of the small fragments are below FISH resolution. Instead, PCF 17a mapped to two human chromosomes and the SFs were above FISH resolution, as such it was manually broken following human alignments in the regions with the lowest adjacency score produced by RACA. Finally, PCF24 was homologous to the entire human chromosome 18 (HSA18), but FISH data indicates that HSA18 corresponds to camel chromosomes 30 and 24. However, we were not able to separate the two fragments.

Then, taking into account the high karyotype stability in all camelid species (Balmus et al., 2007) we used the alpaca physical map (Avila et al., 2014) to assess the internal structure of the PCFs. A total of 52 alpaca genes successfully mapped to 26 camel PCFs (Supplementary Table S2). Although 12 PCFs contained only one gene of the set, it allowed us to confirm their correct placement into camel chromosomes. At least two genes mapped to 15 PCFs, allowing us to orient and assess their structure. Two PCFs (PCF 6b and 2c_3a_16a) disagreed with the alpaca gene map and were manually broken (Supplementary Tables S2, S3). By using the alpaca gene map with enough marker information, we identified these two more disagreements not detected with the FISH data only; therefore, by integrating two physical maps we produced a more reliable assembly (Supplementary Figure S1).

**FIGURE 4:**
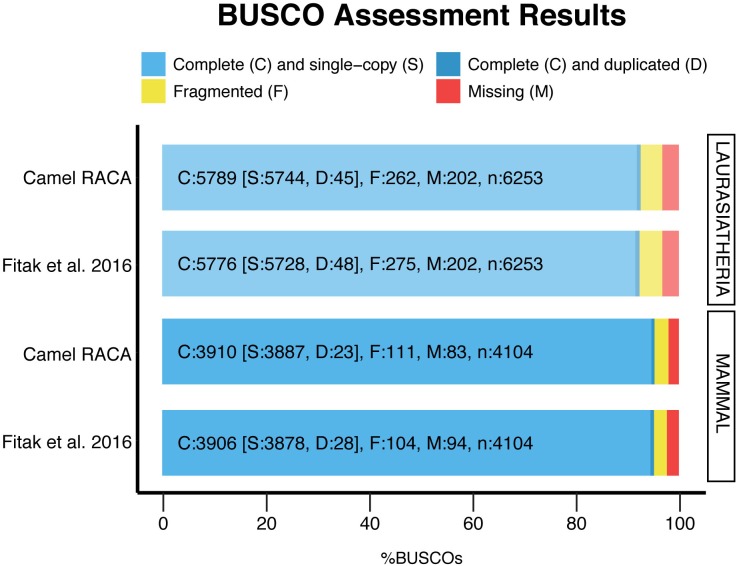
Genome assembly evaluation. The BUSCO dataset of the mammalia odb9 including 4,104 BUSCOs, and the laurariatheria odb9, with 6,253 BUSCOs, were used to assess the new assembly (camel RACA) and the original scaffold-based assembly (Fitak et al., 2016).

After verifying the PCFs and correcting the misassemblies, we used both physical maps to place and orient the PCFs into camel chromosomes (Supplementary Table S3). In doing so, more than 80% of chromosomes were assembled into three camel PCFs: 12 chromosomes were presented by a single PCF, 13 by two PCFs, and four by three PCFs (Table 3). Five camel chromosomes were represented by more than three PCFs, while two chromosomes (CDR24 and CDR30) remained within the PCF24 as we were not able to break it. Then, we assessed the assembly contiguity using the BUSCO (Simão et al., 2015) with two sets of orthologous genes (Figure 4). The newly improved assembly contains more complete single copy BUSCOs and less fragmented genes in both the mammalian and laurariatherian sets, showing an increase of contiguity. Finally, REAPR (Hunt et al., 2013) was used to identify assembly errors without the need of a reference genome (Supplementary Table S4 and Supplementary Figure S2). Overall, we achieved a final dromedary camel chromosome-level assembly by combining *in silico* reconstructions with physical maps.

**Table 3:** Number of PCFs per camel chromosome.

No. PCFs	No. chromosomes	% chromosomes
1	12	33.3
2	13	36.1
3	4	11.1
>3	5	13.9
Unknown	2	5.6


*Further details can be found in Supplementary Table S3.*

## Discussion

In this study, we upgraded the previously published fragmented dromedary camel genome assembly to nearly chromosome-level using a combination of *in silico* chromosome reconstructions, PCR-verification and supporting data from camel and alpaca physical maps. This approach has been previously applied to mammalian genomes, such as the Tibetan antelope (Kim et al., 2013) (2*n* = 60), red fox (Rando et al., 2018) (2*n* = 34), and avian species, including pigeon and peregrine falcon (Damas et al., 2017), and showed high consistency when compared with third-generation sequencing methodologies (Holt et al., 2018). Our approach resulted in a remarkable reduction in fragmentation of the original dromedary assembly by 25-fold, and an N50 increase 35-fold. Compared to other mammalian genomes assembled using the same approach, RACA produced 72 PCFs for dromedary camel, while 60 and 128 PCFs were obtained for Tibetan antelope and red fox, respectively (Kim et al., 2013; Rando et al., 2018). These differences could be explained by three main factors, the initial fragmentation of the scaffold-based assembly, the choice of reference genome and the chromosome rearrangement rate of the phylogenetic clade. Dromedary camel original assembly has an N50 of 1.40 Mb, while Tibetan antelope scaffold N50 was 2.76 Mb, indicating that a higher N50 of the input assembly could reduce the number of PCFs obtained by RACA. Moreover, the divergence time between the Tibetan antelope and the chosen reference genome (cattle) is 24 MY, whereas the divergence time between dromedary camel and cattle is 64.2 MY, suggesting that choosing a reference closely related to the target species improves continuity of RACA assemblies. But this hypothesis does not hold for red fox results, since the fox scaffold-based assembly had an N50 of 11.8 Mb and dog was used as reference genome (with 14 MY divergence time). However, canid lineage is characterized by a high chromosome rearrangement rate including multiple chromosome fissions (Graphodatsky et al., 2000); while cetartiodactyl clade, specially camelids, show a more stable karyotype (Balmus et al., 2007). For RACA, greater similarity between genome structures of the target and reference genomes clearly improves PCF assembly. Thus, a way to further improve the camel assembly would be to use a phylogenetically closer reference genome, e.g., the alpaca genome currently being assembled.

Although the RACA and PCR approach produces reliable assemblies when compared to third generation sequencing methodologies (Holt et al., 2018), we validated the PCFs using previously published physical maps of FISH using human probes on camel chromosomes (Balmus et al., 2007) and alpaca gene mapping (Avila et al., 2014). Our PCF assembly, FISH map, and alpaca marker genes map were highly consistent, with only eight discrepancies, four of which were too small to be detected by FISH (<3 Mb) and did not contain any marker genes. Only two disagreements were above FISH resolution and guided by FISH and alpaca marker genes we corrected one of them. The remaining one consisted of a PCF orthologous to the entire HSA18 and BTA24. However, as shown by FISH and the alpaca gene map, HSA18 is orthologous to two camel chromosomes (CDR24 and CDR30), but we were not able to split it because not enough marker genes from the alpaca set mapped to this PCF. Therefore, comparing PCFs to such data was important, because it allowed us to check whether our assembly was consistent with independent FISH results, perform further verification, and order PCFs along camel chromosomes.

Although placing the PCFs into chromosomes is important to the usability of the dromedary camel genome, more work is required to improve it further. Integrating spatial and sequence information simultaneously, by using Hi-C (Lieberman-Aiden et al., 2009) and/or optical mapping will resolve the inconsistencies we found between FISH and PCFs as well as assemble the PCFs into complete chromosomes. Moreover, sequencing technologies being able to resolve repetitive regions [such as PacBio and Oxford Nanopore (Jain et al., 2018)] will greatly improve the assembly and close the remaining gaps. However, all these approaches are expensive and might not be within the reach of communities working with livestock species in developing countries. Furthermore, the new approaches are not free from limitations, e.g., HiC could result in false rearrangements to be introduced within chromosomes or even errors in joining chromosomes together. Our assembly, therefore, could be used to flag such inconsistently assembled regions and eventually help resolving them. That is why our improved dromedary camel genome assembled at nearly chromosome level is a step forward to a high-quality camel assembly. Moreover, it will facilitate efficient association of phenotype to genotype studies (Glazer et al., 2015) fostering genomic research in camelid species and also inform research on evolution and speciation through chromosomal changes. Furthermore, the methodology used in this study is significantly cheaper compared to many NGS sequencing methods, allowing for lower-income projects to participate in research.

## Author Contributions

DR performed the analysis and drafted the manuscript. DR and MF interpreted the results. DR, DL, and MF wrote the final version of the manuscript.

## Conflict of Interest Statement

The authors declare that the research was conducted in the absence of any commercial or financial relationships that could be construed as a potential conflict of interest.

## Supplementary Material

Figure S1 Camel chromosomes. Blue blocks indicate positive (+) orientation of tracks compared with the camel chromosome while red blocks, negative (-) orientation. Numbers inside each block represent cattle and human chromosomes or dromedary scaffold IDs. Adjacency scores are shown on the right-hand side of the PCF.

Figure S2 Dotplot showing the alignment of our new assembly compared to a previous dromedary camel assembly (Wu et al., 2014).

Table S1 Polymerase chain reaction results and decision made regarding putative chimeric joints in dromedary camel assembly.

Table S2 Alpaca genes mapped in dromedary camel PCFs.

Table S3 Placement of PCFs into dromedary camel chromosomes on the basis of FISH and BAC markers.

Table S4 Assessment of the quality of the new assembly using REAPR.
