# Supplementary material for: A Near Chromosome Assembly of the Dromedary Camel Genome
Source: Front Genet. 2019 Feb 5;10:32. doi: 10.3389/fgene.2019.00032 (PMC6371769; doi:10.3389/fgene.2019.00032)
Supplement: Figure S1 — Camel chromosomes. Blue blocks indicate positive (+) orientation of tracks compared with the camel chromosome while red blocks, negative (-) orientation. Numbers inside each block represent cattle and human chromosomes or dromedary scaffold IDs. Adjacency scores are shown on the right-hand side of the PCF. [file Image_1.pdf]

1a

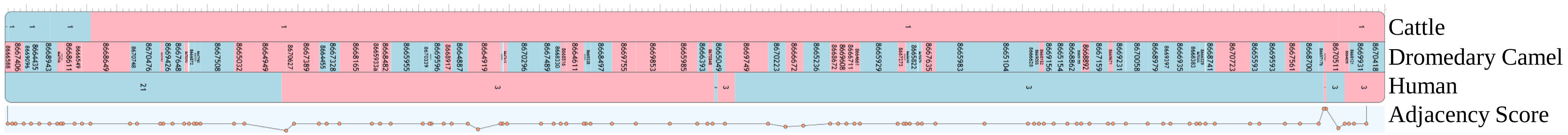

1b

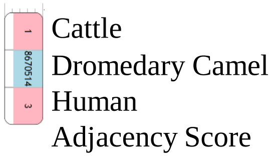

1c

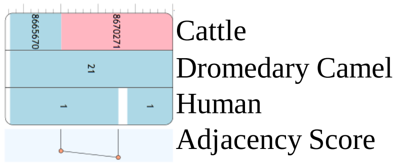

8665670
8670271
21
1
1
Cattle
Dromedary Camel
Human
Adjacency Score

2a

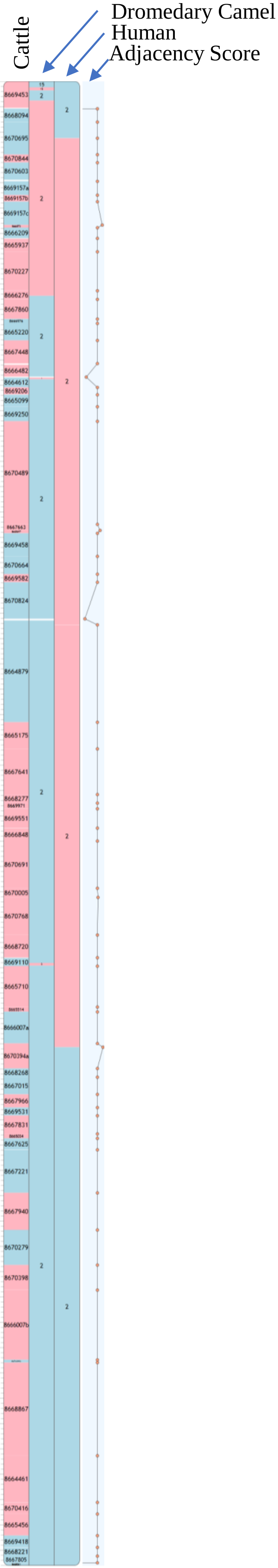

Cattle
Dromedary Camel
Human
Adjacency Score
8669453
8668094
8670695
8670844
8670603
8669157a
8669157b
8669157c
8666209
8665937
8670227
8666276
8667860
8665220
8667448
8666482
8664612
8669206
8665099
8669250
8670489
8669458
8670664
8669582
8670824
8664879
8665175
8667641
8668277
8669551
8666848
8670691
8670005
8670768
8668720
8669110
8665710
8666007a
8670394a
8668268
8667015
8667966
8669531
8667831
8667625
8667221
8667940
8670279
8670398
8666007b
8668867
8664461
8670416
8665456
8669418
8668221
8667805

2b

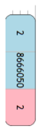

Cattle
Dromedary Camel
Human
Adjacency Score

2C

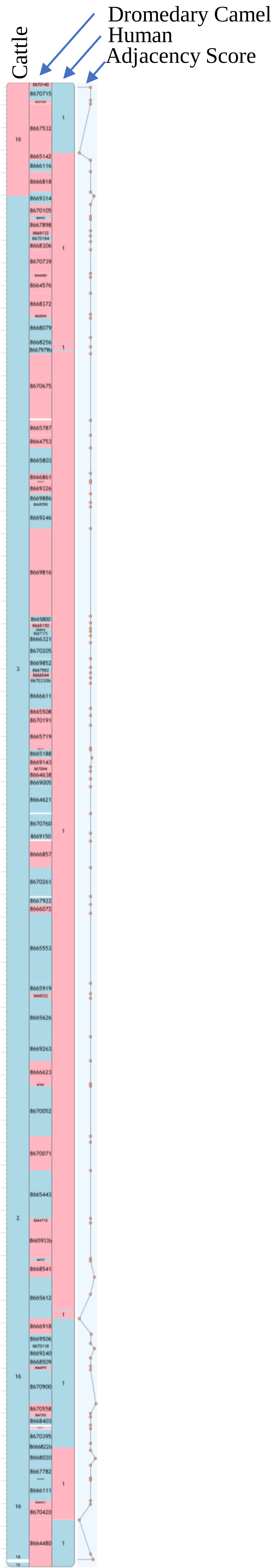

3b

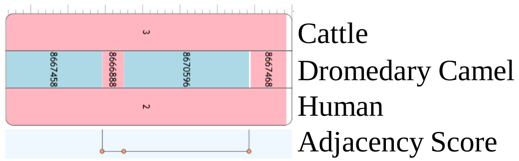
Cattle
Dromedary Camel
Human
Adjacency Score
3
2
8667458
8666888
8670596
8667468

4a_22b

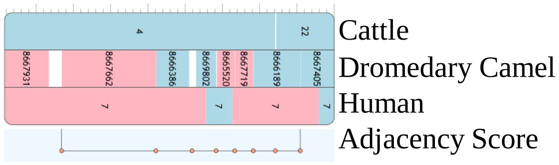

Cattle
Dromedary Camel
Human
Adjacency Score
4
22
8667931
8667662
8666386
8669802
8665520
8667719
8666189
8667405
7
7
7
7

4b

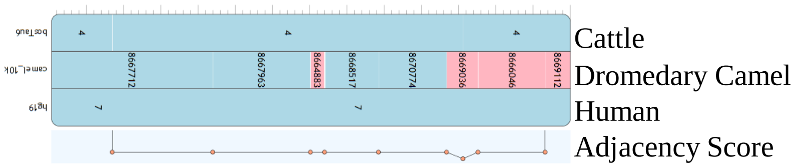
Cattle
Dromedary Camel
Human
Adjacency Score
bosTau6
camel_10k
hg19
4
4
4
7
7
8667712
8667963
8664883
8668517
8670774
8669036
8666046
8669112

4c

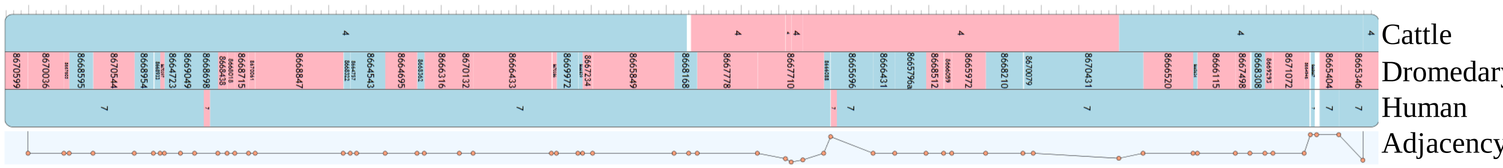
Cattle
Dromedary
Human
Adjacency
8665346
8665404
8671072
8669293
8668308
8667498
8666115
8666520
8670431
8670079
8668210
8665972
8666059
8668512
8665796a
8666431
8665696
8666088
8667710
8667778
8668168
8665849
8667234
8669972
8666433
8670132
8666316
8668362
8664695
8664543
8664737
8668322
8668847
8670061
8668715
8668018
8668438
8668698
8669049
8664723
8668923
8668954
8670544
8668595
8667603
8670036
8670599

4d

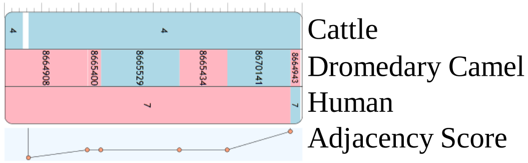

Cattle
Dromedary Camel
Human
Adjacency Score
4
4
8664908
8665400
8665529
8665434
8670141
8664943
7
7

4e

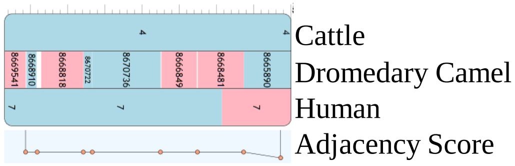
Cattle
Dromedary Camel
Human
Adjacency Score
4
4
8669541
8668910
8668818
8670722
8670736
8666849
8668481
8665890
7
7
7

4fe

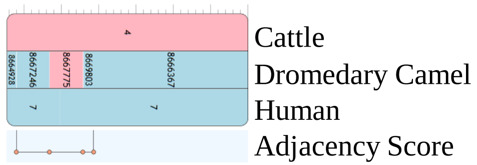

Cattle
Dromedary Camel
Human
Adjacency Score
4
8664928
8667246
8667775
8669803
8666367
7
7

5a

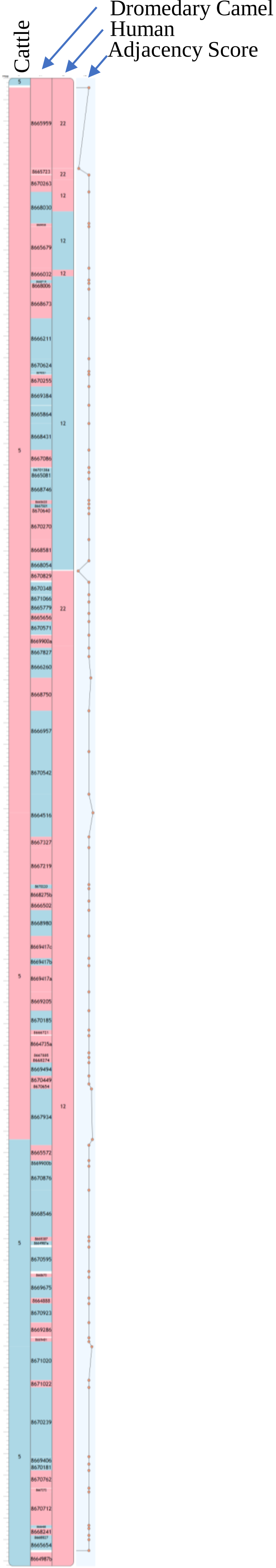

5b

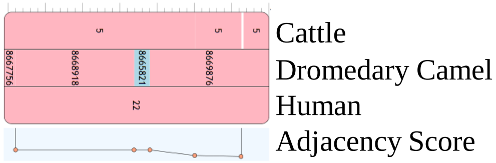

5
5
5
8667756
8668918
8665821
8669876
22
Cattle
Dromedary Camel
Human
Adjacency Score

6a

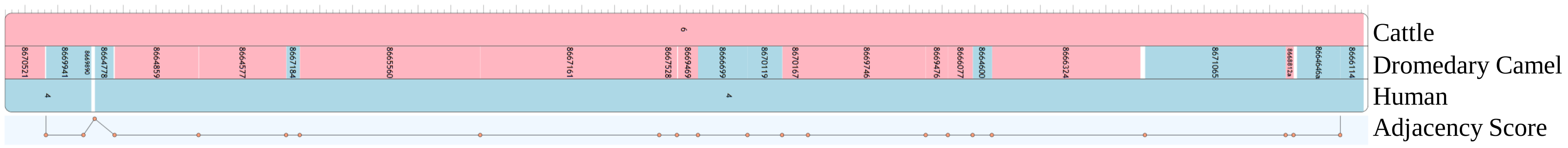

Cattle
Dromedary Camel
Human
Adjacency Score
6
4
4
8670521
8669941
8669890
8664778
8664859
8664577
8667184
8665560
8667161
8667528
8669469
8666699
8670119
8670167
8669746
8669476
8666077
8664600
8666324
8671065
8668812a
8664646a
8666114

6b

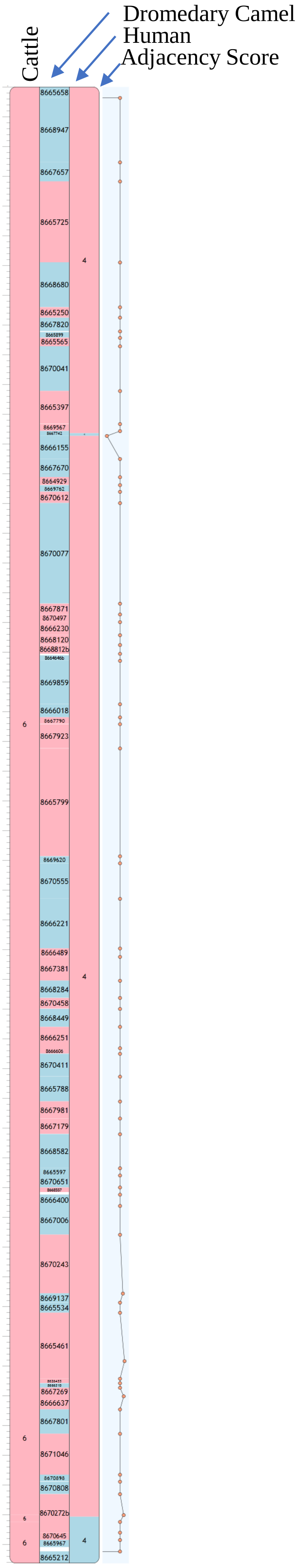

Dromedary Camel
Human
Adjacency Score
Cattle
8665658
8668947
8667657
8665725
4
8668680
8665250
8667820
8665565
8670041
8665397
8669567
8666155
8667670
8664929
8669762
8670612
8670077
8667871
8670497
8666230
8668120
8668812b
8669859
8666018
6
8667790
8667923
8665799
8669620
8670555
8666221
8666489
8667381
4
8668284
8670458
8668449
8666251
8670411
8665788
8667981
8667179
8668582
8665597
8670651
8666400
8667006
8670243
8669137
8665534
8665461
8667269
8666637
8667801
6
8671046
8670898
8670808
8670272b
6
8670645
8665967
6
4
8665212

6c

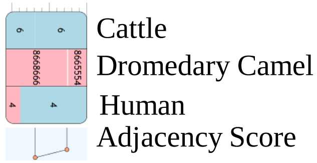
Cattle
Dromedary Camel
Human
Adjacency Score
6
6
8665554
8668666
4
4

6d

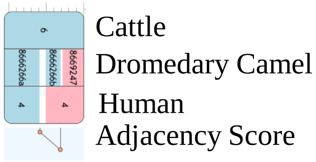
Cattle
Dromedary Camel
Human
Adjacency Score
6
8669247
8666266b
8666266a
4
4

7a_10a_20a

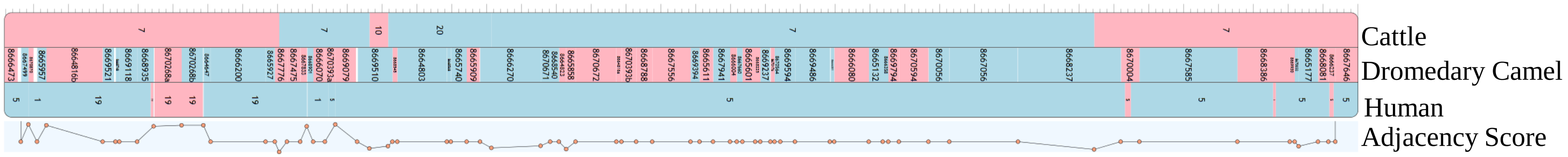

Cattle
Dromedary Camel
Human
Adjacency Score
7
10
20
8666473
8665957
8664816b
8669521
8669118
8668935
8670268a
8670268b
8666200
8665927
8667776
8667475
8666070
8670393a
8669079
8669510
8664803
8665740
8665909
8666270
8670671
8668540
8665858
8670672
8670393b
8668788
8667556
8669394
8665611
8667941
8665601
8669237
8669594
8669486
8666080
8665132
8669794
8670594
8670056
8667056
8668237
8670004
8667585
8668386
8665177
8668081
8667646
5
1
19

7b_10b_20b

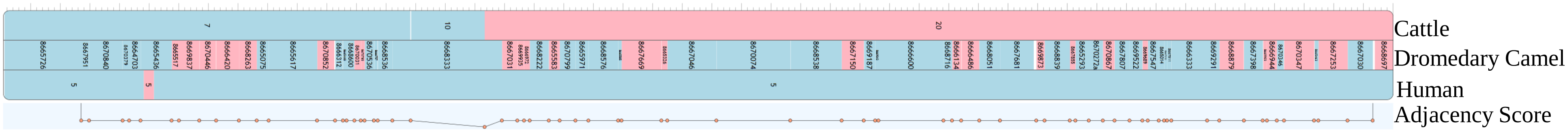

Cattle
Dromedary Camel
Human
Adjacency Score
7
10
20
5
5
5
8665726
8667951
8670840
8670379
8664703
8665436
8665517
8669837
8670446
8666420
8668263
8665075
8665617
8670852
8666312
8668600
8670031
8670536
8668536
8668333
8667031
8669935
8666972
8668222
8665583
8670799
8665971
8668576
8667669
8665326
8667046
8670074
8668538
8667150
8669187
8666600
8668716
8666134
8666486
8668051
8667681
8669873
8668839
8667855
8665293
8670272a
8670867
8667807
8669522
8669689
8667547
8665014
8666333
8669291
8668879
8667398
8666944
8670346
8670347
8667253
8667030
8668697

8a

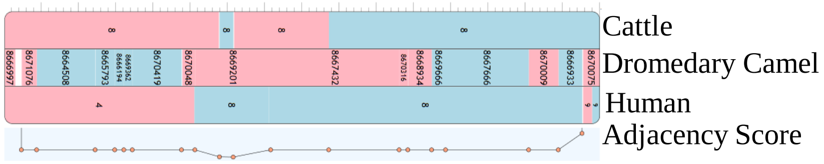
Cattle
Dromedary Camel
Human
Adjacency Score
8
8
8
8
8666997
8671076
8664508
8665793
8669362
8666194
8670419
8670048
8669201
8667432
8670316
8668934
8669666
8667666
8670009
8666933
8670075
4
8
8
9
9

8b

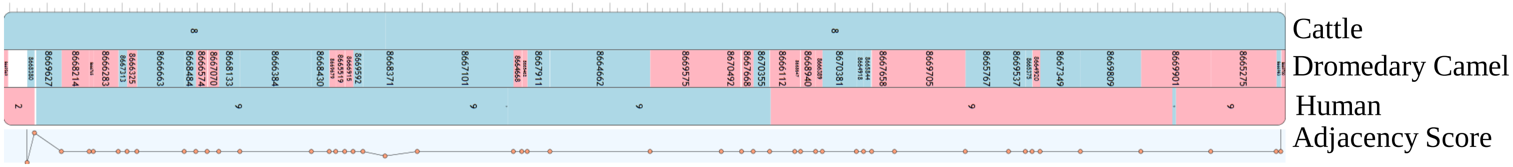
Cattle
Dromedary Camel
Human
Adjacency Score
8
8
9
9
9
9
2
8665275
8669901
8669809
8667349
8664920
8669537
8665767
8669705
8667658
8670381
8668940
8666112
8670355
8667668
8670492
8669575
8664662
8667911
8664668
8667101
8668371
8669592
8666915
8665519
8668430
8666384
8668133
8667070
8666574
8668484
8666663
8666325
8667313
8666283
8668214
8669627
8668380

8c

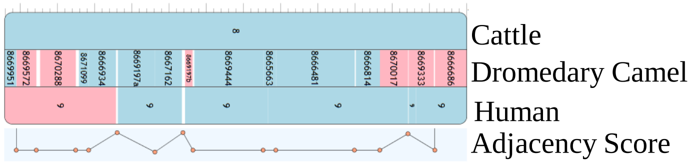

Cattle
Dromedary Camel
Human
Adjacency Score
8
8666686
8669333
8670017
8666814
8666481
8665663
8669444
8669197b
8667162
8669197a
8666934
8671099
8670288
8669572
8669951
9

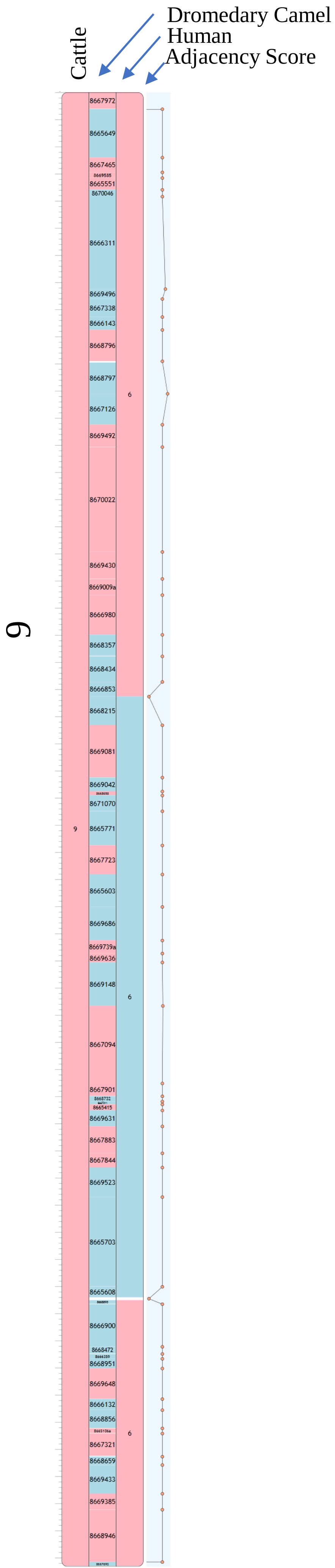

Dromedary Camel
Human
Adjacency Score
Cattle
9
8667972
8665649
8667465
8665551
8670046
8666311
8669496
8667338
8666143
8668796
8668797
8667126
8669492
8670022
8669430
8669009a
8666980
8668357
8668434
8666853
8668215
8669081
8669042
8671070
8665771
8667723
8665603
8669686
8669739a
8669636
8669148
8667094
8667901
8665415
8669631
8667883
8667844
8669523
8665703
8665608
8666900
8668472
8668951
8669648
8666132
8668856
8667321
8668659
8669433
8669385
8668946
6
9
6
6

10c

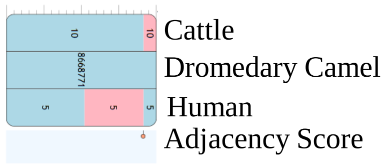

10
10
8668771
5
5
5
Cattle
Dromedary Camel
Human
Adjacency Score

10d

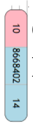
10
8668402
14
Cattle
Dromedary Camel
Human
Adjacency Score

10e_21a

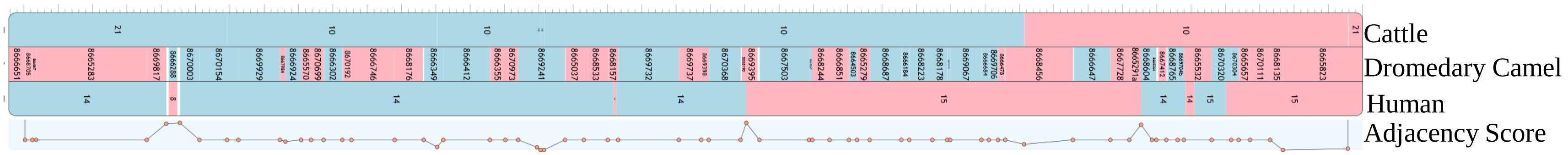

10f_21b

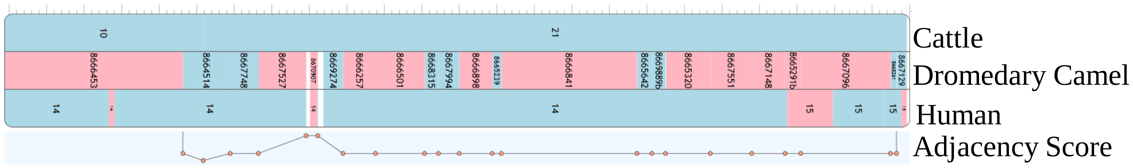

Cattle
Dromedary Camel
Human
Adjacency Score
21
10
8667129
8667096
8665291b
8667148
8667551
8665320
8669889b
8665642
8666841
8665239
8666898
8667994
8668315
8666501
8666257
8669274
8667527
8667748
8664514
8666453
15
15
15
14
14
14

10g

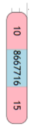
10
8667716
15
Cattle
Dromedary Camel
Human
Adjacency Score

11a

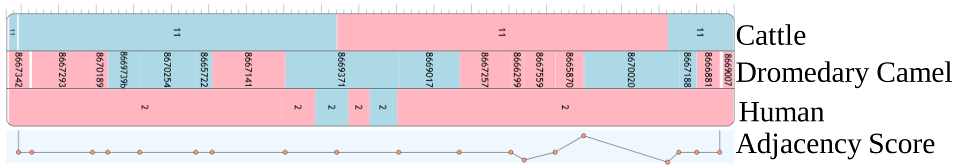

Cattle
Dromedary Camel
Human
Adjacency Score
11
11
11
11
8669007
8666881
8667188
8670020
8665870
8667559
8666299
8667257
8669017
8669371
8667141
8665722
8670254
8669739b
8670189
8667293
8667342
2
2
2
2
2
2

11b

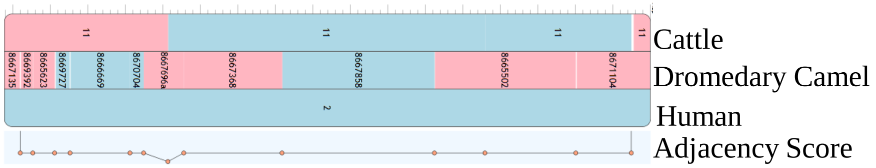

Cattle
Dromedary Camel
Human
Adjacency Score
11
11
11
11
8667135
8669392
8665623
8669727
8666669
8670704
8667696a
8667368
8667858
8665502
8671104
2

11c

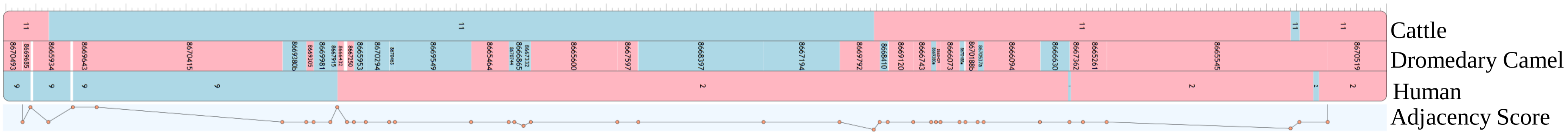
Cattle
Dromedary Camel
Human
Adjacency Score
11
11
11
11
11
8670519
8665545
8665261
8667362
8666630
8666094
8670188b
8666073
8666743
8669120
8668410
8669792
8667194
8668397
8667597
8665600
8667332
8666865
8665464
8669549
8670294
8665953
8667250
8666432
8667915
8669981
8669305
8669380b
8670415
8669643
8665934
8669685
8670493
2
2
2
9
9
9
9

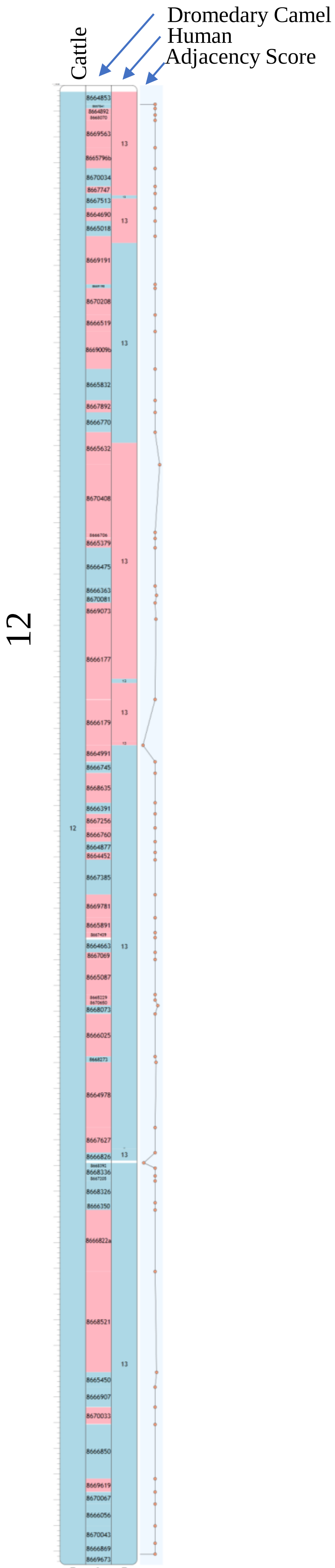
Dromedary Camel
Human
Adjacency Score
Cattle
12

13a

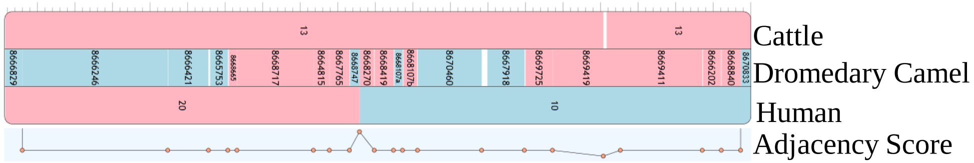

Cattle
Dromedary Camel
Human
Adjacency Score
13
13
8666829
8666246
8666421
8665753
8668665
8668717
8664815
8667765
8668747
8668270
8668419
8668107a
8668107b
8670460
8667918
8669725
8669419
8669411
8666202
8668840
8670833
20
10

13b

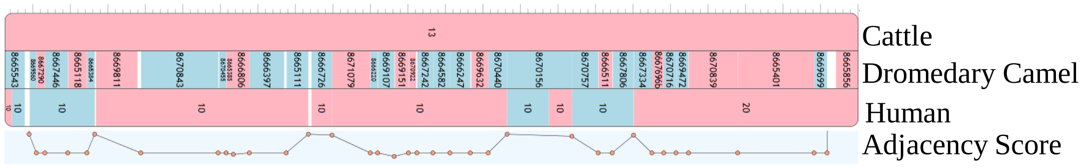
Cattle
Dromedary Camel
Human
Adjacency Score
13
8665856
8669699
8665401
8670839
8669472
8670716
8667696b
8667334
8667806
8666511
8670757
8670156
8670440
8669632
8666247
8664582
8667242
8669151
8669107
8671079
8666726
8665111
8666397
8666806
8670843
8669811
8665118
8667446
8665543
20
10

13c

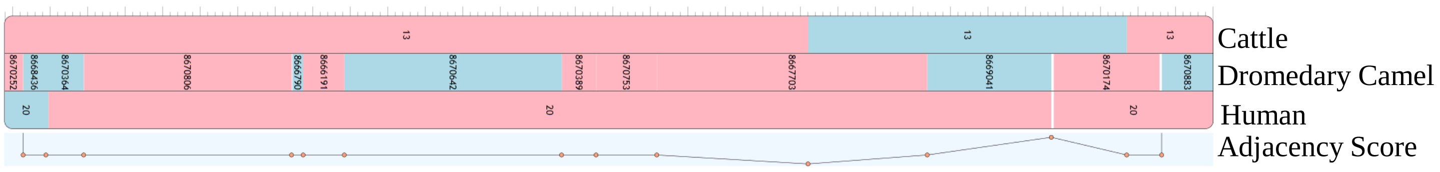

Cattle
Dromedary Camel
Human
Adjacency Score
13
13
13
8670252
8668436
8670364
8670806
8666790
8666191
8670642
8670389
8670753
8667703
8669041
8670174
8670883
20
20
20

14a

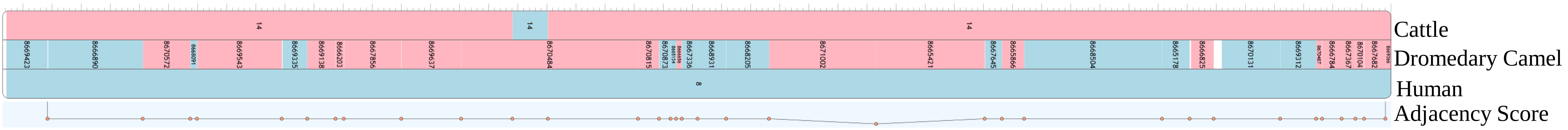
Cattle
Dromedary Camel
Human
Adjacency Score
14
14
14
8
8667682
8670104
8667367
8666784
8669312
8670131
8666825
8665178
8668504
8665866
8667645
8665421
8671002
8668205
8668931
8667336
8670873
8670815
8670484
8669637
8667856
8666203
8669138
8669335
8669543
8668091
8670572
8666890
8669423

14b

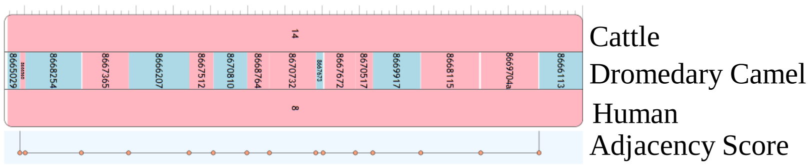
Cattle
Dromedary Camel
Human
Adjacency Score
14
8
8665029
8668254
8667365
8666207
8667512
8670810
8668764
8670732
8667672
8670517
8669917
8668115
8669704a
8666113

15b

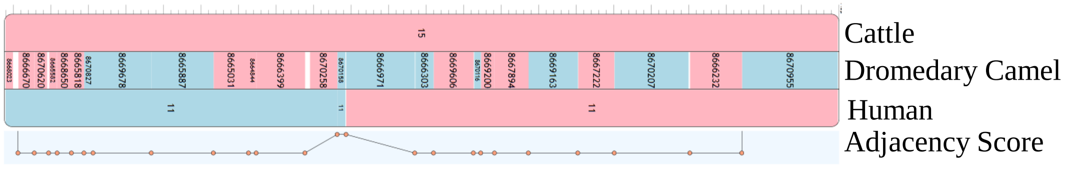
Cattle
Dromedary Camel
Human
Adjacency Score
15
8670955
8666232
8670207
8667222
8669163
8667894
8669200
8670116
8669606
8666303
8666971
8670158
8670258
8666399
8664844
8665031
8665887
8669678
8670827
8665818
8668650
8665582
8670620
8666670
8668023
11
11
11

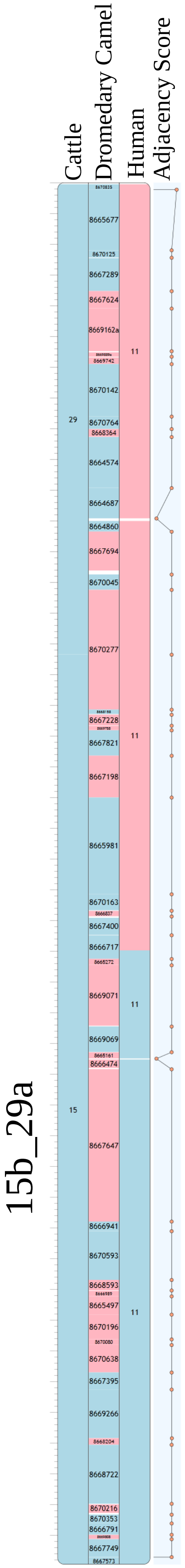
15b_29a
Cattle
Dromedary Camel
Human
Adjacency Score
29
15
11
11
11
11
8665677
8670125
8667289
8667624
8669162a
8669742
8670142
8670764
8668364
8664574
8664687
8664860
8667694
8670045
8670277
8667228
8667821
8667198
8665981
8670163
8667400
8666717
8669071
8669069
8665161
8666474
8667647
8666941
8670593
8668593
8665497
8670196
8670638
8667395
8669266
8668204
8668722
8670216
8670353
8666791
8667749
8667573

15c_29b

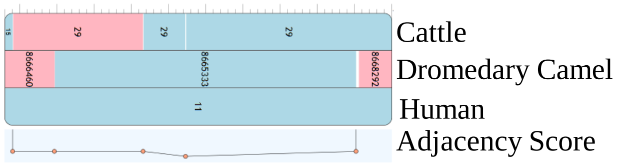

15
29
29
29
8666460
8665333
8668292
11
Cattle
Dromedary Camel
Human
Adjacency Score

16b

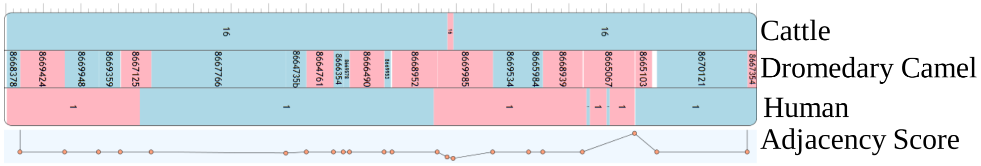

Cattle
Dromedary Camel
Human
Adjacency Score
16
16
8667354
8670121
8665103
8665067
8668939
8665984
8669534
8669985
8668952
8666490
8666354
8664761
8664735b
8667766
8667125
8669359
8669948
8669424
8668378

16c

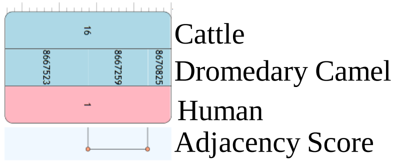

16
8667523
8667259
8670825
1
Cattle
Dromedary Camel
Human
Adjacency Score

17a

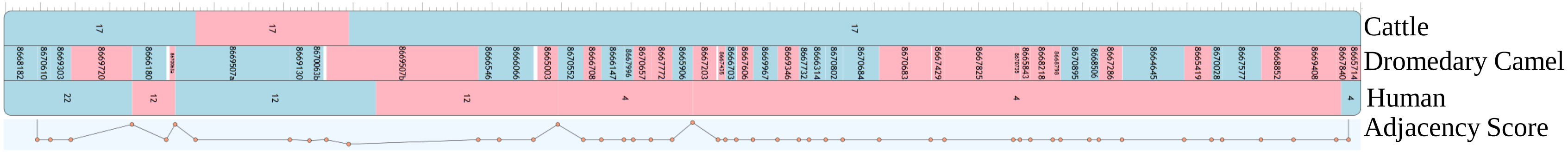

Cattle
Dromedary Camel
Human
Adjacency Score
17
17
17
22
12
12
12
4
4
4
8668182
8670610
8669303
8669720
8666180
8669507a
8669130
8670063b
8669507b
8666546
8666066
8665003
8670552
8666708
8666147
8667996
8670657
8667772
8665906
8667203
8666703
8667606
8669967
8669346
8667732
8666314
8670802
8670684
8670683
8667429
8667825
8665843
8668218
8670895
8668506
8667286
8664645
8665419
8670028
8667577
8668852
8669408
8667840
8665714

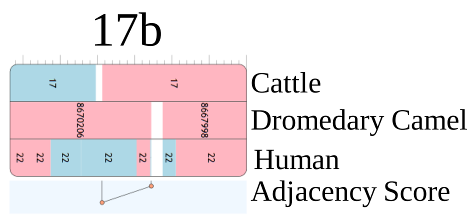
17b
17
17
8670206
8667998
22
22
22
22
22
22
22
Cattle
Dromedary Camel
Human
Adjacency Score

18a

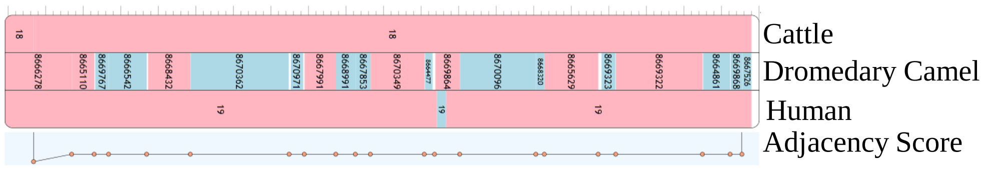

Cattle
Dromedary Camel
Human
Adjacency Score
18
18
8666278
8665110
8669767
8666542
8668432
8670362
8670971
8667991
8668991
8667853
8670349
8669864
8670096
8665629
8669323
8669322
8664861
8669868
19
19
19

18b

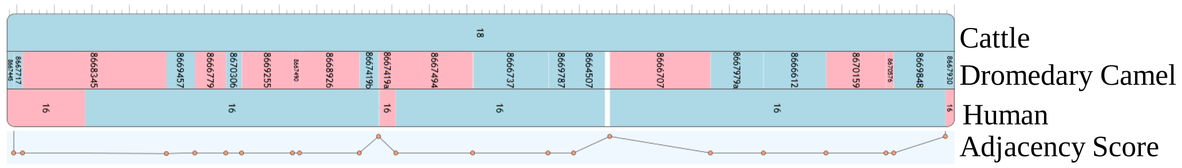

Cattle
Dromedary Camel
Human
Adjacency Score
18
8667717
8667445
8668345
8669457
8666779
8670306
8669255
8667490
8668926
8667419b
8667419a
8667494
8666737
8669787
8664507
8666707
8667979a
8666612
8670159
8670576
8669848
8667930
16

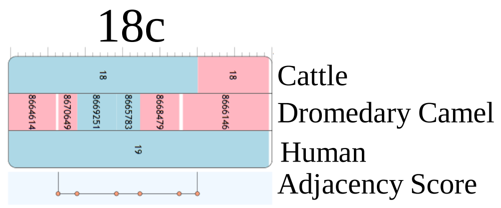
18c
Cattle
Dromedary Camel
Human
Adjacency Score
18
18
8664614
8670649
8669251
8665783
8668479
8666146
19

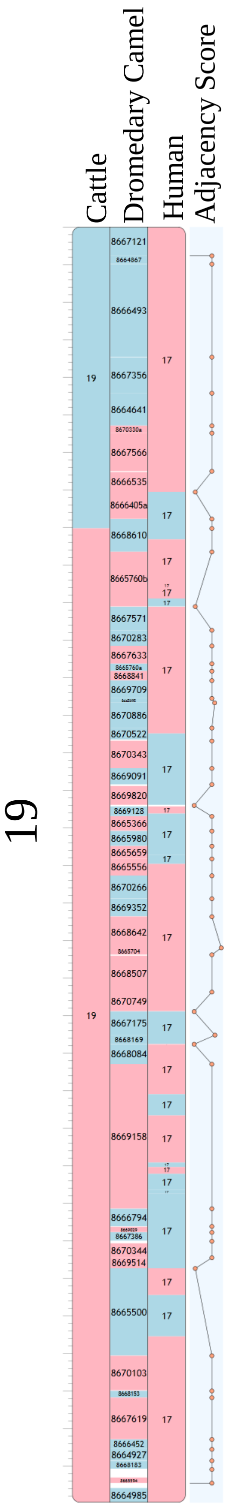
19
Cattle
Dromedary Camel
Human
Adjacency Score
8667121
8666493
8667356
8664641
8667566
8666535
8666405a
8668610
8665760b
8667571
8670283
8667633
8668841
8669709
8670886
8670522
8670343
8669091
8669820
8669128
8665366
8665980
8665659
8665556
8670266
8669352
8668642
8668507
8670749
8667175
8668169
8668084
8669158
8666794
8667386
8670344
8669514
8665500
8670103
8667619
8666452
8664927
8668183
8664985
17

21c

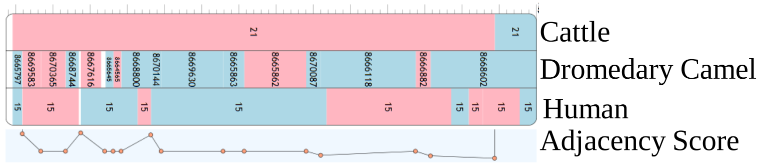
Cattle
Dromedary Camel
Human
Adjacency Score
21
21
8665797
8669583
8670365
8668744
8667616
8668800
8670144
8669630
8665863
8665862
8670087
8666118
8666882
8668602
15

21d

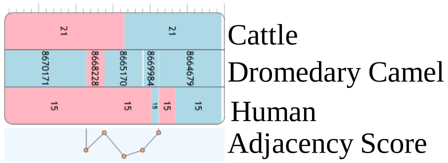
Cattle
21
21
Dromedary Camel
8670171
8668228
8665170
8669984
8664679
Human
15
15
15
15
Adjacency Score

22b

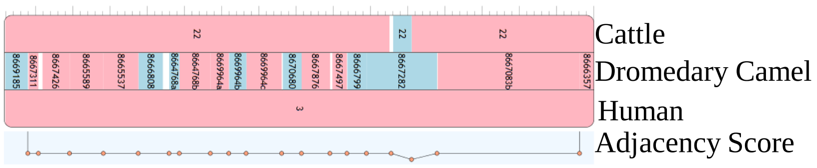
Cattle
Dromedary Camel
Human
Adjacency Score
22
22
22
8666357
8667083b
8667282
8666799
8667497
8667876
8670680
8669964c
8669964b
8669964a
8664768b
8664768a
8666808
8665537
8665589
8667426
8667311
8669185
3

22c

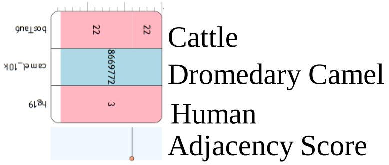

Cattle
Dromedary Camel
Human
Adjacency Score
bosTau6
camel_10k
hg19
22
22
8669772
3

22d

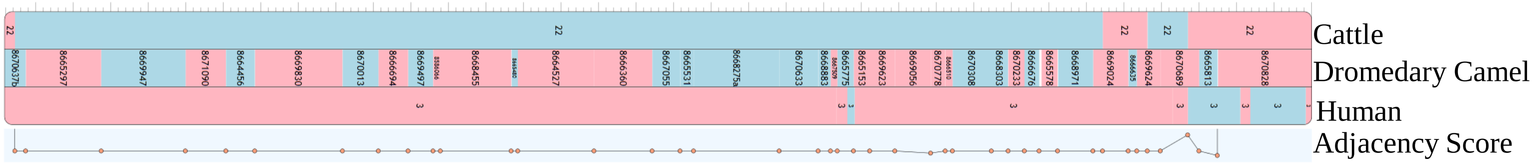
Cattle
Dromedary Camel
Human
Adjacency Score
22
8670637b
8665297
8669947
8671090
8664456
8669830
8670013
8666694
8669497
8668455
8664527
8666360
8667055
8665531
8668275a
8670633
8668883
8665775
8665153
8669623
8669056
8670778
8670308
8668303
8670233
8666676
8665578
8668971
8669024
8669624
8670689
8665813
8670828
3

23a

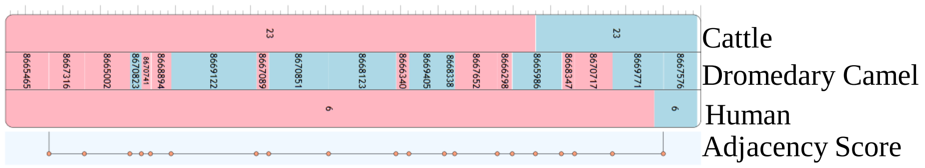
Cattle
Dromedary Camel
Human
Adjacency Score
23
23
6
6
8667576
8669771
8670717
8668347
8665986
8666298
8667652
8668338
8669405
8666340
8668123
8670851
8667089
8669122
8668894
8670741
8670823
8665002
8667316
8665465

23b

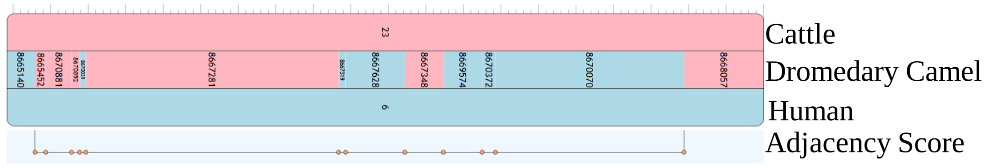
23
6
8665140
8665452
8670881
8667281
8667628
8667348
8669574
8670372
8670070
8668057
Cattle
Dromedary Camel
Human
Adjacency Score

24

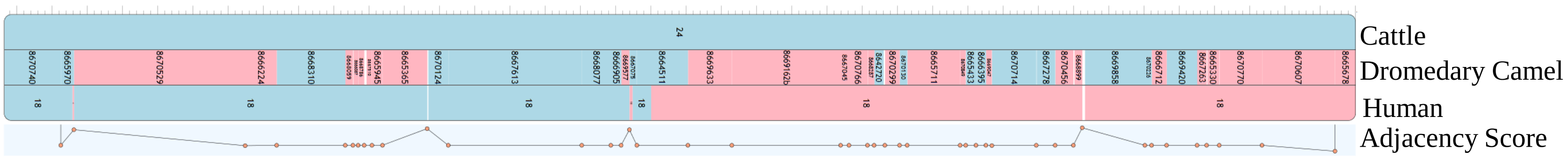

Cattle
Dromedary Camel
Human
Adjacency Score
24
8670740
8665970
8670529
8666224
8668310
8668059
8665945
8665365
8670124
8667613
8668077
8666905
8669577
8667075
8664511
8669633
8669162b
8667045
8670766
8642720
8670299
8670130
8665711
8665433
8666395
8670714
8667278
8670456
8668899
8669858
8670226
8666712
8669420
8667263
8665330
8670770
8670607
8665678
18
18
18
18
18
18

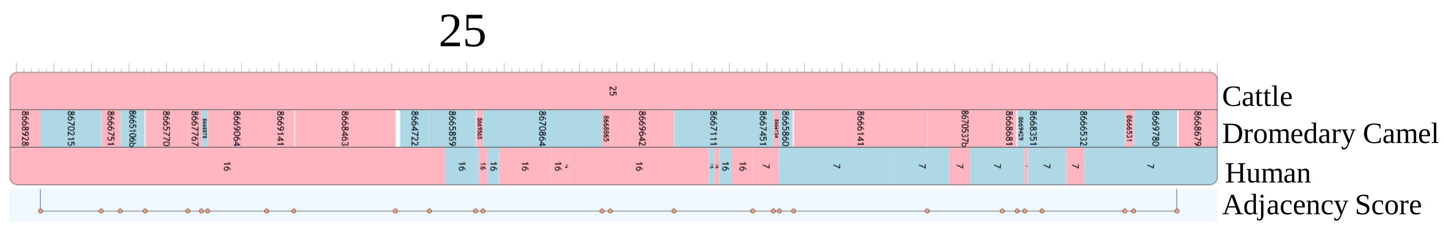

25
Cattle
Dromedary Camel
Human
Adjacency Score
8668679
8669780
8666532
8668351
8668681
8670537b
8666141
8665860
8667451
8667111
8669642
8670864
8665859
8664722
8668463
8669141
8669064
8667767
8665770
8665106b
8666751
8670215
8668928
7
16

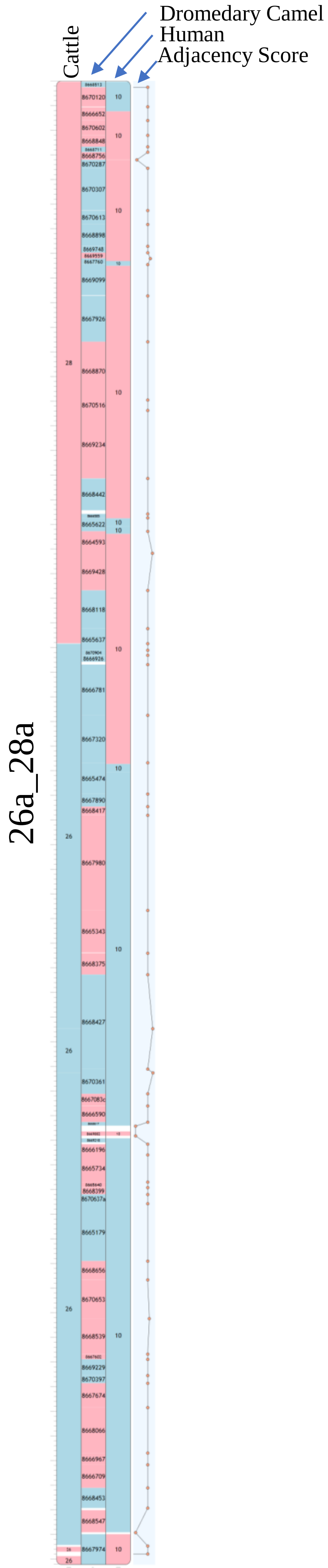

Dromedary Camel
Human
Adjacency Score
Cattle
26a_28a

26b

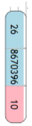

Cattle
Dromedary Camel
Human
Adjacency Score

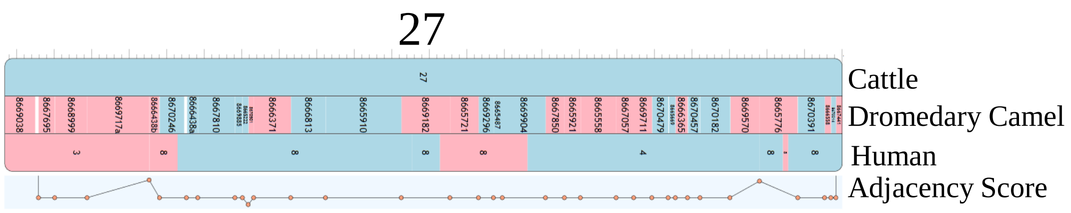
27
Cattle
Dromedary Camel
Human
Adjacency Score
27
8670391
8665776
8669570
8670182
8670457
8666365
8670479
8669711
8667057
8665558
8665921
8667850
8669904
8665487
8669296
8665721
8669182
8665910
8666813
8666371
8667810
8666438a
8670246
8666438b
8669717a
8668999
8667695
8669038
8
8
4
8
8
8
8
3

28b

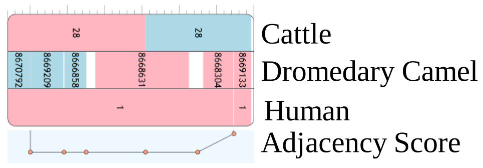
Cattle
Dromedary Camel
Human
Adjacency Score
28
28
8670792
8669209
8666858
8668631
8668304
8669133
1
1

28c

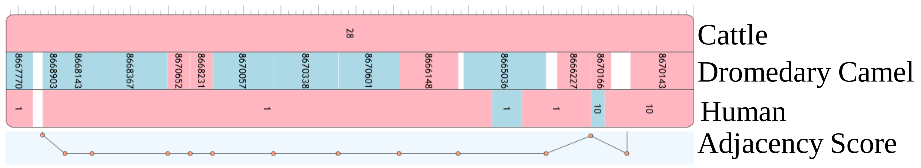
Cattle
Dromedary Camel
Human
Adjacency Score
28
8667770
8668903
8668143
8668367
8670652
8668231
8670057
8670338
8670601
8666148
8665036
8666227
8670166
8670143
1
1
1
1
10
10

29c

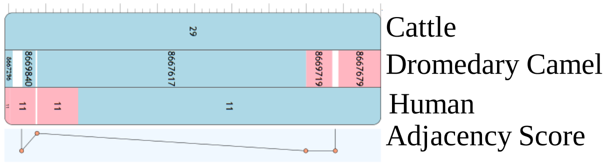
Cattle
Dromedary Camel
Human
Adjacency Score
29
8669840
8667617
8669719
8667679
11
11
11
