# Supplementary figures and images for: A Near Chromosome Assembly of the Dromedary Camel Genome
Source: Front Genet. 2019 Feb 5;10:32. doi: 10.3389/fgene.2019.00032 (PMC6371769; doi:10.3389/fgene.2019.00032)

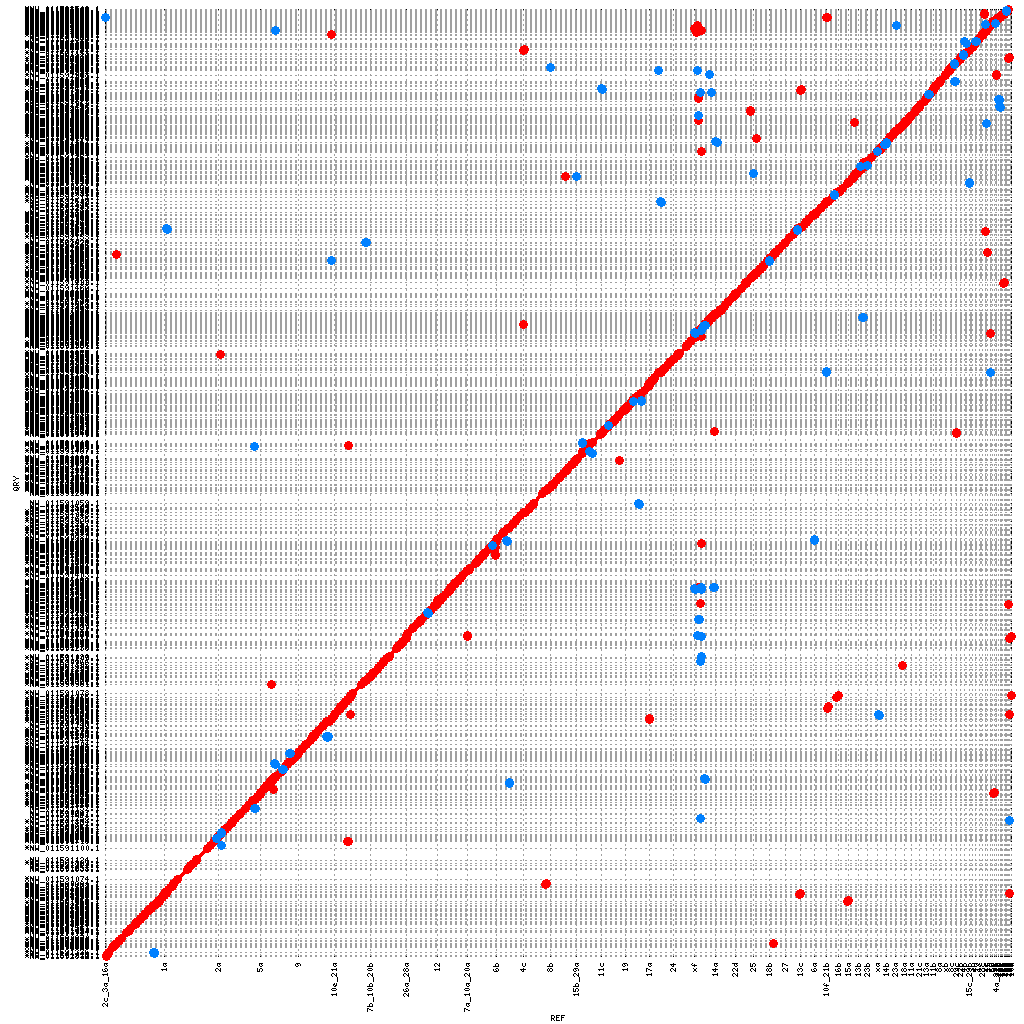

Supplement: Figure S2 — Dotplot showing the alignment of our new assembly compared to a previous dromedary camel assembly (Wu et al., 2014). [file Image_2.png]
