# Supplementary material for: A Near Chromosome Assembly of the Dromedary Camel Genome
Source: Front Genet. 2019 Feb 5;10:32. doi: 10.3389/fgene.2019.00032 (PMC6371769; doi:10.3389/fgene.2019.00032)
Supplement: Table S1 — Polymerase chain reaction results and decision made regarding putative chimeric joints in dromedary camel assembly. [file Table_1.docx]

**Supplementary Table S1. PCR results and decision made regarding putative chimeric joints in dromedary camel assembly.**

| **Scaffold IDs** | **Cattle Chr1** | **Cattle Chr2** | **Physical coverage** | **PCR**  **Round** | **PCR result*** | **Decision** |
| --- | --- | --- | --- | --- | --- | --- |
| 8666350/8666822b | 12 | 12 | 5.5 | 2 | - | inconclusive |
| 8669889a/8669162b | 29 | 21 | 5.5 | 2 | + | chimeric |
| 8669009a/8666519 | 9 | 12 | 6.5 | 2 | - | inconclusive |
| 8669889a/8665642 | 29 | 21 | 10.1 | 2 | - | inconclusive |
| 8669739a/8669686 | 9 | 9 | 12.7 | 2 | - | inconclusive |
| 8665933 | 1 | 2 | 13.5 | 1 | + | not chimeric |
| 8667083a/8666357 | X | 22 | 15.8 | 2 | + | chimeric |
| 8670138 | 5 | X | 16.5 | 1 | + | not chimeric |
| 8669380 | 11 | 11 | 16.5 | 1 | + | not chimeric |
| 8665796 | 4 | 12 | 18.7 | 1 | + | not chimeric |
| 8670330a/8664641 | 19 | 3 | 19.9 | 2 | - | inconclusive |
| 8664827/8667083b | X | 22 | 20.1 | 2 | - | inconclusive |
| 8664735a/8666721 | 5 | 5 | 20.2 | 2 | - | inconclusive |
| 8666822a/8668020 | 12 | 16 | 22.1 | 2 | + | chimeric |
| 8664735b/8667766 | 16 | 16 | 22.5 | 2 | - | inconclusive |
| 8670189/8669739b | 9 | 11 | 24.8 | 2 | + | chimeric |
| 8666405a/8664719 | 19 | X | 25.2 | 2 | + | chimeric |
| 8670637a/8665179 | 26 | 26 | 27.0 | 2 | - | inconclusive |
| 8670716/8667696b | 11 | 13 | 40.9 | 2 | + | chimeric |
| 8667696a/8667368 | 11 | 13 | 43.8 | 2 | - | inconclusive |
| 8669704a/8668765 | 14 | 10 | 46.2 | 2 | - | inconclusive |
| 8669964a/8669964b | 22 | 22 | 184.7 | 2 | + | chimeric |
| 8669417 | 5 | 5 | **212.5^**^** | 1 | + | not chimeric |
| 8669157 | 2 | 2 | 214.4 | 1 | + | not chimeric |
| 8670188 | 11 | 11 | 216.0 | 1 | + | not chimeric |
| 8665760 | 19 | 19 | 232.5 | 1 | + | not chimeric |
| 8666007 | 2 | 2 | 245.5 | 1 | + | not chimeric |
| 8668812 | 6 | 6 | 250.5 | 1 | + | not chimeric |
| 8669417 | 5 | 5 | 267.0 | 1 | + | not chimeric |
| 8667979 | 18 | 3 | 291.4 | 1 | + | not chimeric |
| 8664768 | 22 | 22 | 328.7 | 1 | + | not chimeric |
| 8665291 | 10 | 21 | 329.7 | 1 | + | not chimeric |

*From PCR round I, only scaffolds with positive results are shown, as SFs with negative results were retested in run II, where their RACA-suggested alternative order and adjacency were tested. Lower case letters next to scaffold IDs represent fragments of scaffolds.

**Underlined is the PCR-established physical coverage threshold (212.5) above which PCR results become consistent with camel original assembly scaffold structures.
