# Supplementary material for: A Near Chromosome Assembly of the Dromedary Camel Genome
Source: Front Genet. 2019 Feb 5;10:32. doi: 10.3389/fgene.2019.00032 (PMC6371769; doi:10.3389/fgene.2019.00032)
Supplement: Table S4 — Assessment of the quality of the new assembly using REAPR. [file Table_4.docx]

**Supplementary Table S4. Assessment of the quality of the new assembly using REAPR.**

Total length: 1,886,430,519

Number of sequences: 72

Mean sequence length: 26,200,423.88

Length of longest sequence: 122,837,231

N50 = 54364184, n = 13

N60 = 50112088, n = 16

N70 = 37237037, n = 21

N80 = 23126555, n = 27

N90 = 15262262, n = 37

N100 = 206421, n = 72

Number of gaps: 114,119

Total gap length: 25,957,214

Error free bases: 87.97%
